# 异硫氰酸酯抗肿瘤作用机制研究新进展

**DOI:** 10.3779/j.issn.1009-3419.2017.03.11

**Published:** 2017-03-20

**Authors:** 会敏 王, 克 徐

**Affiliations:** 300052 天津，天津医科大学总医院，天津市肺癌研究所，天津市肺癌转移与肿瘤微环境重点实验室 Tianjin Key Laboratory of Lung Cancer Metastasis and Tumor Microenvironment, Tianjin Lung Cancer Institute, Tianjin Medical University General Hospital, Tianjin 300052, China

**Keywords:** 异硫氰酸酯, 抗肿瘤, 凋亡, 细胞周期阻滞, 转移, 自噬, Isothiocyanate, Anti-tumor activity, Apoptosis, Cell cycle arrest, Metastasis, Autophagy

## Abstract

异硫氰酸酯（isothiocyanate, ITC）是由硫代葡萄糖苷经黑芥子酶水解形成的天然小分子，广泛存在于十字花科蔬菜中。大量研究证明异硫氰酸酯可以通过诱导肿瘤细胞周期阻滞、促进肿瘤细胞凋亡、诱导活性氧的产生等多种机制抑制肿瘤细胞在体内、体外的增殖。近期研究发现异硫氰酸酯还可以抑制肿瘤细胞侵袭转移、诱导内质网应激和细胞自噬。本文对异硫氰酸酯的抗肿瘤作用机制进行综述。

## Abstract (en)

Isothiocyanates (ITCs) are naturally occurring small molecules that are generated by the enzymic hydrolysis of glucosinolate in cruciferous vegetables. Numerous studies showed that ITCs inhibit the growth of tumors by the mechanisms including inducing cell cycle arrest, promoting apoptosis and producing reactive oxygen species *in vitro* and *in vivo*. Recent studies showed that ITCs also inhibit metastasis of cancer cells, induce endoplasmic reticulum stress and autophagy. This review summarizes the antitumor mechanisms of ITCs.

2015年我国癌症新发病例数及死亡人数分别为429.2万例和281.4万例，世界卫生组织（World Health Organization, WHO）曾发布报告称，中国大陆的癌症发病率已经处于世界首位^[1]^。因此，癌症的预防与治疗刻不容缓。流行病学研究表明增加蔬菜的摄入能够降低部分癌症发生的风险^[2, 3]^。十字花科蔬菜中含有丰富的硫代葡萄糖苷（glucosinolate），硫代葡萄糖苷含有共同的化学结构R-C(=N-O-SO_3_-)-S-glucose。目前已知超过120种硫代葡萄糖苷是稳定并且水溶性的，这也为后续的研究提供了一定的基础^[4]^。十字花科蔬菜经咀嚼或组织破坏后释放黑芥子酶（myrosinase），黑芥子酶水解硫代葡萄糖苷形成异硫氰酸酯R-N=C=S。根据R侧链的不同异硫氰酸酯分为三种不同的类型：芳香族、脂肪族以及吲哚族^[5]^。目前用于研究的异硫氰酸酯主要有萝卜硫素（sulforaphane, SFN），异硫氰酸苯乙酯（phenethyl isothiocyanate, PEITC），异硫氰酸苄酯（benzyl isothiocyanate, BITC）和异硫氰酸丙烯酯（allyl isothiocyanate, AITC）。其中PEITC是目前唯一到临床试验阶段的异硫氰酸酯^[6]^。大量研究表明异硫氰酸酯能够很好地预防肿瘤的发生，并且在体内、体外很多研究中异硫氰酸酯都表现了很好的抗肿瘤特性。所以，本文主要针对以上几种异硫氰酸酯在肿瘤的预防以及抗肿瘤方面的研究新进展进行综述，并进一步探讨异硫氰酸酯的抗肿瘤作用。

## 1 异硫氰酸酯对肿瘤的预防作用

大量研究^[7-10]^发现摄入十字花科蔬菜能够有效地预防胰腺癌、卵巢癌、结肠癌、前列腺癌等癌症的发病风险。在致癌因素的刺激下，异硫氰酸酯能够抑制Ⅰ相酶（phase Ⅰ enzymes）如细胞色素P450（cytochrome P450）的活性，阻止致癌物前体的激活^[11]^。另外，异硫氰酸酯能够激活Ⅱ相酶（phase Ⅱ enzymes），使得Ⅰ相反应产物与葡萄糖醛酸（glucuronic acid）以及谷胱甘肽（glutathione, GSH）结合，促使致癌物尽快排出体外^[12]^。

Wu等^[13]^研究发现，摄入十字花科蔬菜使得非吸烟女性肺癌的发病风险降低41%，并且改变肺癌患者预后。Yuan等^[14]^研究发现，PEITC能够抑制烟草特定致癌物4-(methylnitrosamino)-1-(3-pyridyl)-1-butanone（NNK）的活化代谢。Crampsie等^[15]^研究发现PEITC作用于小鼠肺腺癌模型后，肝脏和肺组织的P450活性降低，葡萄糖醛酸转移酶和谷胱甘肽-S-转移酶活性也明显升高。并且PEITC还能够抑制烟草致癌物NNK导致的DNA甲基化，是很好的肿瘤预防物质。

## 2 异硫氰酸酯的抗肿瘤作用

### 2.1 抑制肿瘤细胞增殖

#### 2.1.1 诱导ROS产生

活性氧簇（reactive oxygen species, ROS）是机体氧化代谢过程中产生的高活性含氧自由基或过氧化物，过多的ROS能够直接或间接地损伤细胞内核酸、蛋白质、脂质等大分子物质的生理功能。ROS与细胞的生长分化以及炎症反应等相关，是很多疾病发生的诱因^[16]^。Wu等^[17]^研究发现ITCs能够诱导产生ROS，并且PEITC和BITC还能够降低GSH的水平（GSH是细胞内重要的抗氧化剂），进一步增加了ROS的累积；加入抗氧化剂后ROS的产生得到抑制。Yeh等^[18]^研究发现ROS累积能够导致DNA损伤，并激活p53以及其下游*p21*和*Bax*基因，抑制肿瘤细胞增殖。Chou等^[19]^研究发现PEITC通过诱导脑胶质瘤细胞产生ROS，激活caspase凋亡途径，从而诱导细胞凋亡。核转录因子Nrf2（NF-E2-related factor 2）在抗氧化反应中起到重要作用，其受胞浆蛋白Keap1的负调控，当发生氧化应激反应时，细胞内的Nrf2与Keap1解离，并与Maf结合形成异二聚体，进一步激活抗氧化反应元件（antioxidant response element, AREs）介导的下游基因的表达^[20]^。Hu等^[21]^研究发现PEITC能够通过调控Nrf2诱导氧化应激，抑制肿瘤细胞增殖。另外，Gao等^[22]^研究发现PEITC还能够抑制乳腺癌细胞线粒体呼吸链复合物Ⅲ的活性，降低耗氧率，诱导ROS产生，并进一步激活JNK信号通路，最终导致细胞凋亡。Wu等^[17]^研究结果显示在非小细胞肺癌（non-small cell lung cancer, NSCLC）中，ITCs能够通过诱导ROS的产生调控转移相关基因的表达，抑制高转移肺癌细胞侵袭转移。

#### 2.1.2 诱导肿瘤细胞周期阻滞

肿瘤细胞长期处于快速的增殖阶段，即S期+G_2_期，当增殖阶段肿瘤细胞的比例越高时肿瘤细胞增殖越快。大量研究表明ITCs能够通过诱导细胞周期阻滞来抑制肿瘤细胞增殖^[23, 24]^。Chen等^[25]^研究发现PEITC能够诱导口腔癌细胞G_0_期/G_1_期细胞周期阻滞，并通过线粒体途径诱导细胞凋亡。Chou等^[19]^研究发现脑胶质瘤细胞经PEITC作用后G_1_期比例显著增加，并且细胞形态发生改变，细胞存活率降低，且呈时间和剂量依赖性。Wu等^[23]^研究发现在骨原性肉瘤中，异硫氰酸酯剂量不同诱导发生的细胞周期阻滞不同，5 μM BITC能够诱导G_2_期/M期阻滞，10 μM BITC能够诱G_0_期/G_1_期阻滞；PEITC分别为5 μM和7.5 μM时能够诱导G_2_期/M期阻滞，而10 μM PEITC能够诱导S期细胞周期阻滞。Yan等^[26]^研究也发现BITC和PEITC能够通过调节cyclin B1蛋白的表达诱导肺癌细胞G_2_期/M期细胞周期阻滞。

#### 2.1.3 抑制肿瘤血管新生

肿瘤的生长离不开血管新生，如果没有新生的血管来提供足够的营养，肿瘤生长将被抑制。Xiao等^[27]^研究发现PEITC能够通过抑制Akt信号通路的激活来抑制毛细血管样网状结构的生成，并且抑制间接体内实验中新生血管的生成。另外，AITC和PEITC能够通过下调一氧化氮（nitric oxide, NO）和肿瘤坏死因子α（tumour necrosis factor-α, TNF-α）来抑制血管生成^[28]^。Gupta等^[29]^研究发现，在缺氧环境中PEITC能够抑制神经脑胶质瘤细胞内缺氧诱导因子（hypoxia inducible factor-1α, HIF-1α）和血管内皮生长因子（vascular endothelial growth factor, VEGF）的表达，并且激活ERK和Akt信号通路，从而抑制肿瘤血管的生成。另外，在前列腺癌细胞、胶质瘤细胞、结肠癌细胞、肝癌细胞和乳腺癌细胞中PEITC均可以抑制HIF-1α的累积，抑制VEGF、表皮生长因子（epidermal growth factor, EGF）以及粒细胞集落刺激因子（granulocyte colony-stimulating factor, G-CSF）的分泌，下调VEGF-R2蛋白的表达，进一步抑制肿瘤细胞的增殖。

### 2.2 诱导肿瘤细胞凋亡

细胞凋亡是指细胞的程序性死亡，是细胞的基本生物学现象。细胞的主要凋亡途径有两条：一是线粒体凋亡途径。当细胞受到凋亡因子刺激时，Bcl-2蛋白家族Bak、Bax激活并结合到线粒体外膜，使得线粒体外膜透化，细胞色素C由线粒体释放到胞质，引起线粒体膜电位发生改变，并激活caspase凋亡途径。Lee等^[30]^研究发现PEITC能够下调Bcl-2和XIAP抗凋亡蛋白，上调p53表达，使得Bax由胞质转移到线粒体，促进粒体细胞色素C和凋亡诱导因子的释放，激活caspase凋亡途径，促进PARP裂解，从而诱导细胞凋亡。二是死亡受体凋亡途径。死亡受体属于肿瘤坏死因子受体超家族，当死亡受体与相应的配体结合后，其结构发生改变，经过一系列的信号转导过程，激活下游caspase信号途径，诱导细胞凋亡。肿瘤坏死因子相关凋亡诱导配体（tumor necrosis factor-related apoptosis-induced ligand, TRAIL）在抗肿瘤中起重要作用。Lee等^[31]^研究发现PEITC与TRAIL共同作用后，能够敏化对TRAIL耐受的恶性胶质瘤细胞，诱导ROS累积，激活死亡受体5（death receptor 5, DR5），从而诱导细胞凋亡。体内研究结果也表明，PEITC与TRAIL共同作用比单独作用能够更好地抑制肿瘤的生长。

另外，研究^[32]^发现ITCs还能通过其他的信号途径来诱导肿瘤细胞的凋亡。在肺癌细胞中，BITC通过抑制Akt/MAPK信号通路激活，诱导肺癌细胞凋亡。PEITC还可以通过p38MAPK/JNK途径增加DNA损伤结合蛋白2（DNA damage binding protein 2, DDB2）的表达，诱导肿瘤细胞的凋亡，抑制肿瘤细胞增殖^[33]^。Xiao等^[34]^研究发现PEITC可以通过诱导DNA损伤，线粒体功能紊乱，导致前列腺癌细胞凋亡。内质网是真核细胞内蛋白质合成的重要细胞器，内质网内环境稳定是其发挥功能的重要条件。近年来，越来越多的研究发现内质网应激在细胞凋亡中起到重要作用。Zhang等^[35]^研究发现BITC能够诱导肺癌细胞发生内质网应激。

### 2.3 抑制肿瘤细胞侵袭转移

肿瘤的侵袭转移是癌症患者死亡的主要原因。正常细胞通过细胞之间的黏附分子（cell adhesion molecule, CAM），如上皮钙粘素（E-cadherin）紧密的黏着在一起。而肿瘤细胞之间E-cadherin减少，使得肿瘤细胞之间的黏附力减弱，易于分离。游离肿瘤细胞与基底膜附着，溶解细胞外基质（extracellular matrix, ECM），突破基底膜，从而发生局部侵袭。Ho等^[36]^研究发现BITC可以抑制基质金属蛋白酶（matrix metalloproteinase, MMP）-2的mRNA转录水平，还能够降低MMP-2、MMP-7、MMP-9和uPA（uPA在基底膜的分解中起到重要的作用）等侵袭转移相关蛋白的表达，进一步激活ERK、PKCs/MAPK和PI3K/Akt/NF-κB等信号通路来抑制胃癌细胞侵袭转移。Gong等^[37]^研究发现PEITC能够抑制STAT3的激活，进而抑制前列腺癌细胞侵袭转移。另外，本课题组前期研究也发现BITC和PEITC能够通过激活MAPK信号通路诱导高转移性NSCLC细胞凋亡，进而抑制肺癌细胞侵袭转移^[26]^。

### 2.4 诱导细胞自噬

自噬（autophagy）是存在于真核细胞中的高度保守的生命过程，其在细胞应对多种生长压力和环境刺激时，对维持生命体的正常发育等过程均起到关键作用^[38]^。早在1993年日本科学家^[39]^就发现酵母在营养缺乏的时候会出现自噬，并发现第一个自噬基因Atg1，之后对自噬做出了进一步的研究。自噬在维持细胞稳态中起到重要作用，然而过多的细胞自噬可以诱导细胞死亡。由此可见，自噬是一把双刃剑，它既可以促进肿瘤细胞凋亡，又可以对肿瘤细胞起到保护作用^[40]^。

Lin等^[41]^研究发现BITC作用于前列腺癌细胞后，能够抑制自噬负性调节因子mTOR的表达，激活细胞自噬，且当自噬被抑制后BITC能够进一步促进细胞的凋亡，表明由BITC诱导发生的自噬在肿瘤细胞中起到保护的作用。本课题组前期研究也发现BITC作用于肺癌细胞后，能够通过内质网应激诱导保护性自噬的发生^[35]^。然而Bommareddy等^[42]^研究发现PEITC通过调控自噬基因Atg5，激活Akt-mTOR信号通路，诱导前列腺癌细胞发生自噬，进一步抑制肿瘤细胞增殖。FoxO是Forkhead转录因子家族最重要的家族成员，作为PI3K/Akt信号通路的直接下游分子，FoxO1能转录及传导多种生长及细胞因子信号，参与细胞的生长发育、新陈代谢，并且与多种肿瘤的发生发展有着密切关系。Xiao等^[43]^研究发现BITC作用后使得FoxO1乙酰化增加，促使mTOR与其亚基的解离，诱导乳腺癌细胞发生自噬，导致细胞凋亡，从而发挥抗肿瘤活性。总之，异硫氰酸酯能够通过诱导细胞自噬，促进或者抑制肿瘤细胞的增殖，这也为肿瘤的治疗提供了一个新的理念和思路^[44]^。

## 3 其他

异硫氰酸酯还用于与化疗药物联合使用的研究^[45]^。有研究^[46]^发现AITC与放射治疗药物联合使用后，能够增加NSCLC细胞对放射治疗的敏感性。Gupta等^[47]^研究发现PEITC能够特异性诱导HER2阳性的乳腺癌细胞凋亡，抑制肿瘤在体内的生长；PEITC与乳腺癌常规治疗药物阿霉素联合使用后，使得阿霉素药物剂量降低，同时对肿瘤细胞的杀伤作用增强。

MicroRNAs是在真核生物中发现的一类内源性的具有调控功能的非编码RNA。最近研究发现，microRNA的表达与多种癌症相关。Zhang等^[48]^研究发现PEITC作用于前列腺癌细胞后，能够通过调节microRNA-194的表达，下调MMP-2和MMP-9蛋白表达，抑制前列腺癌细胞的侵袭。Basu等^[49]^研究发现BITC可能通过作用于microRNA-221和microRNA-375靶点，抑制胰腺癌细胞的恶性增生。Izzotti等^[50]^研究发现，在香烟环境中，PEITC能够保护肺microRNAs不受香烟环境调控，从而预防肺癌的发生。Wagner等^[51]^研究发现，AITC能够显著降低microRNA-155的水平，同时增加Nrf2的mRNA和蛋白水平，发挥抗炎作用。另外，Slaby等^[52]^研究发现识别microRNAs结合位点可能与ITCs预防结肠癌的发生相关。

## 4 结语

综上所述，异硫氰酸酯作为一个广泛存在于十字花科蔬菜中的天然的小分子，能够预防多种肿瘤的发生。同时异硫氰酸酯对正常的细胞并没有毒性，这也增加了临床应用的可能性。大量研究发现异硫氰酸酯能够通过调节不同的蛋白、不同的信号通路来抑制肿瘤细胞的增殖和侵袭转移，很好的发挥抗肿瘤作用，这也为肿瘤的个性化治疗提供依据。虽然PEITC已经到了临床试验阶段，但是异硫氰酸酯距离临床应用阶段还有很长的路要走，还需大量的临床试验，异硫氰酸酯的抗肿瘤作用机制也需要更加深入的研究。
